# Fatigue Induced by Labour
^1^An address given at the meeting of the Bristol Medico-Chirurgical Society on February 14th, 1917.


**Published:** 1917-07

**Authors:** A. F. Stanley Kent

**Affiliations:** Professor of Physiology, University of Bristol


The Bristol
Medico-Chirurgical Journal.

"*Scire est nescire, nisi id me*
*Scire alius sciret.*"

JULY, 1917.

### FATIGUE INDUCED BY LABOUR.[1]

BY

A. F. STANLEY KENT, M.A., D.Sc., Oxon.,

*Professor of Physiology, University of Bristol.*

The address included an account and demonstration of the methods and apparatus employed in recent researches on industrial fatigue amongst munition and other classes of industrial workers.

---

THE subject of industrial fatigue has long interested me, and experiments had already been undertaken in my laboratory some years before the war began. When that happened the work gained additional interest, in consequence of the bearing which it had on the production of munitions of war. Since then nearly the whole of the energies of three workers have been devoted to the inquiry.

The simplest form of fatigue may be illustrated by taking as an example a physical system which possesses a certain

[1] An address given at the meeting of the Bristol Medico-Chirurgical Society on February 14th, 1917.

amount of energy, and from which energy is lost at a definite rate. Many of us on these dark nights have endeavoured to avoid fellow-passengers in the streets by the aid of pocket flash-lamps, and many have been disappointed to find how short a time they lasted. Such a lamp may be taken as an example. The chemical energy of the cell is gradually dissipated, and the apparatus becomes "fatigued," the fatigue being represented by a simple loss of energy.

In the same way it is possible to demonstrate the dissipation of energy in a muscle. If a muscle be cut from the body and placed in suitable surroundings, it will be found to possess energy, but the amount of energy it can transform is limited. This is illustrated in the experiments upon the table. When fatigued these muscles have lost energy, and in that way resemble the exhausted flash-lamp. But other changes also have taken place, and in addition to being poor in energy, the muscle has become clogged with the chemical products of its own activity—"waste products," as they are called. That these waste products are important in the production of fatigue is shown by an old experiment, where a weak solution of common salt is passed through the blood-vessels of an exhausted muscle, with the result that a remarkable recovery takes place, though from its nature the salt solution is evidently incapable of replenishing the store of energy. The recovery in this case may not unreasonably be attributed to the removal of waste products.

Things are somewhat different when a muscle in the body is considered. Here the circulating blood continually brings new stores of energy, whilst the waste is as continually removed. It might be supposed that fatigue would be postponed indefinitely, and indeed fatigue in its extremest grades is so postponed for the life of the animal, provided that certain necessary conditions be fulfilled. For instance, food must be taken, digested and absorbed, or the blood itself will become starved and no longer capable of nourishing the muscles, and periods of rest must be afforded for the repair of the tissues.

It is evident then that one of the causes of fatigue is the using up of energy, and another, and a very important one, is the accumulation of waste products.

So much for muscle. But the nerve, which is attached to the muscle, has to be considered also. The actual nerve fibre is very resistant to fatigue. It may be stimulated artificially for a long time, and will show but slight signs of change. It is otherwise with the special connecting-link between muscle and nerve, for here there seems to be a mechanism which may be tired easily. But even this is not the weakest link. In a workman turning out munitions of war or other product, we have neither a set of simple muscles, nor of muscles attached to simple nerve fibres carrying on the work. The whole apparatus is under the control of the central nervous system, and as the central nervous system is the most highly organised portion of the mechanism, so it is the most delicate, and the most easily fatigued. It follows that the condition known as industrial fatigue concerns the central nervous system far more closely than it does the nerve fibres or muscles. And since a delicate and highly-organised mechanism is concerned, it is not surprising to find that it is susceptible not only to the influences of activity —the normal producer of fatigue—but also to the influence of a great variety of other agents. Chief amongst these is the poison sometimes produced in the alimentary canal as a result of disturbances of digestion. Persons who suffer from dyspepsia not infrequently show signs of fatigue also. They are tired when they get up in the morning, tired when they go to bed at night, and yet they often lead by no means active lives. It is probably a case of poisoning by toxins absorbed from the intestine, and it is interesting to note that poisons of a somewhat similar nature may be isolated from the blood of a fatigued animal, and when injected into the blood will cause signs of fatigue in an animal which has not been active. Some persons have even gone so far as to assert that not only toxins of fatigue, but antitoxins also may be produced, and that these last, when passed into the blood of a tired animal, are able to abolish the signs of fatigue.

The Italian physiologist Mosso has described an experience which shows how greatly the effects of fatigue may be accentuated when the blood supply is even partially cut off from the central nervous system. He describes the flight of quails, which at certain seasons of the year cross over to Sicily from the opposite coast of Africa, about one hundred and fifty miles distant. On their arrival the brains of these birds had become very anæmic, and as a result of this and their long flight they were quite unable to see. Indeed, on landing they often dashed themselves against trees and other objects in their path, and Mosso asserts that the natives, knowing this, were accustomed to resort to the sea-coast and collect the birds for food.

To come nearer home. The late Sir Lauder Brunton describes an experience of his own whilst he was working in London. After a long day at the hospital he arrived home one night to find a letter awaiting him from the editor of a journal to which he was accustomed to contribute. The editor required an article on a certain subject that night. We all know what it means to do extra work of this kind after a hard day's work, and can sympathise with the author. However, he determined to write the article, and sat down to do so. But he found to his exasperation that no ideas would come. He then began to wonder what the reason could be. Yesterday he could have written the article easily ; his brain to-day was the same as yesterday, only that yesterday he was not tired. And then he thought that perhaps it was because to-day he was tired, and the blood was not circulating through the brain in sufficient volume to refresh the centres, that his ideas refused to flow. Therefore, as the blood could not reach the brain, he determined to bring the brain down to the level of the blood, and this he did by laying his head upon the table. At once he began to write easily, though as soon as he raised his head all ideas left him. The article was accordingly finished with his head resting on the table.

Mr. W. G. Lecky, the historian, was described as having a large, magnificent head on a long and willowy neck. In his case also it is asserted that the circulation was not sufficiently vigorous to carry enough blood to the brain whilst he was in the erect position, and that therefore his famous works were written whilst he lay upon a sofa. The fact apparently is that he did not actually lie upon the sofa, but knelt upon it, and used its broad flat end as a desk.

Probably the popular idea of an American business man sitting in a chair with his feet on the chimney-piece has a similar origin.

Industrial fatigue is more complicated than any of the forms so far mentioned. It depends on muscular and mental fatigue, on worry, bad atmosphere, ill-health and starvation. It varies in amount in different factories, and in the same factory it varies with the type of worker, and with the particular conditions present. But it is a mistake to suppose that it is determined entirely, or even principally, by the conditions inside the mill. The home conditions of the worker, the distance he lives from his work, the nature of his leisure occupations—if any—and, above all, the nature and the adequacy of his food—these things exercise a most important influence upon the character and the grade of fatigue. Factory conditions are important also, but the authorities take care that these shall be at any rate tolerable. They cannot secure fresh air, however, since the workers are often stubbornly set against it, and even where new ventilating apparatus has been installed will see that it is not used. This avoidance of fresh air by the worker is probably due to two causes. Many of them have been brought up to regard open windows with horror, and they are often so insufficiently clothed that free ventilation means discomfort from cold. The result is that a close atmosphere is preferred, and they will often stay in the corner of a stuffy workroom rather than go into a properly-ventilated mess-room for a meal.

In addition to such general causes of a lowered vitality, in many factories there are special conditions connected with the work which are detrimental to health. In a room where cyanide gauze was drying the whole of our party were convinced that they had caught severe colds, and one of them even sought a chemist in order to purchase a remedy. But on the following day all the "colds" had disappeared. Nevertheless, constant exposure to dust capable of producing such effects must have an influence upon the general health, more especially as one of the constituents of the dust is a salt of mercury. Indeed, an examination of the teeth of operatives in the room showed that this was the case.

The manufacture of surgical dressings will be of interest to a medical audience. A good deal of time was spent in investigating conditions in a factory devoted to such work. It may be supposed that the processes were simple, but it must be explained that all the processes from raw cotton to finished dressings of various kinds were carried out in one mill. Some of these processes are as follows: opening, cleaning, carding, spinning, winding, warping, setting-in, slashing or taping, weaving, bleaching, washing, drying, lint making, dyeing, impregnating with antiseptic, compressing, packing. Other works visited were engaged in coal mining, chemical making, engineering, printing, the manufacture of explosives and army stores. Every one of these industries is divided into numerous separate processes, so it is not surprising that the number of individual tests carried out already exceeds forty thousand.

Some of the tasks performed entail heavy labour for girls, and even for men. Other processes involve but little actual labour, but constant watchfulness. Views illustrating the character of the work, and the type of operative employed, are thrown upon the screen.

The actual tests used were for a time a matter of anxiety. Even muscular fatigue requires care for its proper registration; industrial fatigue, depending upon the diverse causes described, is elusive and difficult to detect with certainty. It is still more difficult to estimate and to measure. Moreover, of the tests available for the measurement of fatigue, very few can be used in a factory, where leisure is scanty, habits rough, and workers altogether unaccustomed to scientific methods. The tests selected must be quick, easily carried out, and, in order that wilful interference with results may be avoided, beyond the control of the examinee.

The tests which have been adopted are four in number, viz. measurement of the reaction time, of the acuity of sight, of the acuity of hearing, and of blood pressure. A complicated reaction was selected so that the time measured might be as long as possible; in this way the relative magnitude of the experimental error was diminished. Blood pressure is affected by a great variety of conditions, but when properly safeguarded its measurement gives valuable results.

The factory day is divided up in a different manner in different parts of the country and in different industries. One method is to divide it into four periods, which for description I have called early morning period, morning period, afternoon period, and overtime period. The early morning period begins at 6 a.m. and ends at 8 a.m., there is then half an hour's interval for breakfast. The morning period begins at 8.30 a.m. and ends at 12.30 p.m., there is then an hour's break for dinner. The afternoon period begins at 1.30 p.m. and ends at 5.50 p.m., when there is half an hour's interval for tea. The overtime period in this particular mill begins at 6 p.m. and ends at 8 p.m.

The two slides now shown have been drawn from curves obtained from two different individuals of a group tested by different methods. As will be observed, the form of the two curves is similar, an indication that similar conditions of labour produce in similar individuals similar amounts of fatigue, and that different tests record these in a similar manner. The broad outlines of the fatigue curve are shown also—the fall during the day, indicating development of fatigue, the rise at night, indicating recovery; the gradual decline during the week, indicating the accumulation of fatigue through the carrying over of balances from day to day, the greater effect of an overtime day in producing fatigue, and the lessened resistance towards the end of the week.

The tests made were not confined altogether to the operatives of the factories. Members of the office staff have been tested over a considerable period, and the fact established that here also a large amount of fatigue may exist.

Amongst the workers it was found that the amount of fatigue varied considerably in different periods of the day, and an examination of the curves of output led to the same conclusion. Contrary perhaps to expectation, it is found that the early morning is not the time of greatest activity. The output in this period may be conspicuously bad. In the two middle periods of the day output is good. The reason is not far to seek. In regarding the early morning as a time of great activity, food, warmth, and comfort are pre-supposed. In a factory where work begins at 6 a.m. many workers arrive either unfed, or having taken merely a cup of tea. It is not until the eight o'clock breakfast interval that they take a satisfactory meal. With regard to warmth, the mill is often chilly in the early morning, and does not become comfortably warm until an hour or so later. And it must not be forgotten that many of the hands are badly clothed. The result is the unsatisfactory output mentioned.

In the middle-day periods the conditions are better, and output is correspondingly higher. In the period which is described as overtime, and which is only worked in times of stress, the condition of the worker with regard to fatigue, and the output, are alike bad. A fair day's work has already been done, fatigue has accumulated, rest is due, and powers low. In this period it is significant that a given task will produce greater fatigue than in the earlier periods of the day.

As might be expected, some difference is found between the effects of work performed at night and the ordinary daily task. Night work is more tiring than day work as a rule, but the statement requires to be explained. It is well known that the physiological processes are at their lowest ebb in the early morning, and it is reasonable to expect that a given amount of work performed at that time would result in a maximum amount of fatigue. Where experiments have been made the expectation has been realised, but only at the commencement of a spell of night work. The human organism is extraordinary in its power of adaptation to new conditions, and no sooner are such conditions as those attending night work imposed than adaptation commences. The result is that in a short time the period of minimal physiological activity becomes shifted from the early morning to the early afternoon, and the time at which work is now performed is made to correspond with the time of physiological activity. The body is tuned to a new rhythm, and its performance becomes as efficient as ever. So long as the labour by night and rest by day go on, the physiological reversal will persist, but should the old arrangement be reverted to a new adaptation will be required, and as this will take some days to become complete, a period of inefficiency will appear. It is an example of how ignorance of physiological fact may interfere with efficient organisation, that in many mills efficiency is constantly prejudiced by a weekly change of individuals from night work to day work, and *vice versa*, for it is evident that such frequent change involves constant new adaptations, and as these take some days for their completion, the proportion of days on which efficiency is high is greatly reduced. It is hoped that this cause of inefficiency may be dealt with in the future.

Another factor which tends to upset the regular accumulation of fatigue throughout the week, and which will have to be considered in the future, is that which I have referred to as the "Monday effect." Lassitude and disinclination to work are often present on Monday morning, and diminished efficiency is observed when work is attempted. This condition is found not only in industrial occupations, but also in such as typewriting, knitting, shorthand writing, etc. My experience is that it is marked especially in occupations which involve the learning of complicated processes. It results generally in a poor condition of the worker on Monday morning, improvement towards evening, although work has been performed. Indeed, if no work be performed on Monday the condition of lessened efficiency is transferred to Tuesday, and work attempted on that day shows characters similar to those usually found on Monday. Under ordinary conditions, however, when work has been performed on Monday, and the evening improvement has occurred, the following night's rest, instead of increasing that improvement, will actually be followed by a deterioration, so that the condition is worse on

Tuesday morning than on the previous evening. Afterwards, the ordinary rhythm is observed, each evening showing a worse condition, each morning a condition of improvement.

The occurrence of such inefficiency at the beginning of the week is difficult to explain. It has been attributed to an unwise use of the week-end rest and of the week's wages. But alcohol apparently has nothing to do with it, whilst it occurs equally amongst those of regular and of irregular life. Moreover, something similar is to be seen in the daily variations of efficiency which have already been mentioned. It has been shown that the early morning period is generally a time of low efficiency, and that an improvement occurs as the day wears on. And—a very important point—so far as the Monday effect itself is concerned, it is not well marked in the case of unskilled workers. Thus the evidence points to the real cause of inefficient work on Monday, and in the first working period of any particular day, being traceable, not to any injurious influence acting on the worker, but rather to an abstention from work, resulting in operations which, as a result of practice, are ordinarily performed quickly and well, being to some extent forgotten, and to that extent requiring to be re-learnt before the old efficiency can be regained. It is, in fact, a case of a loss of co-ordination rather than of actual fatigue. The inefficiency often fails to show itself in operatives who are commencing new work, and in untrained workers imported from outside. These individuals, not having learnt the work thoroughly, show little deterioration as a result of the week-end rest. They have less to forget than veteran workers.

If now we consider the changes which occur during the course of a typical week, it will be found that two distinct processes are at work. It is the resultant of these processes that we measure. There is in the first place a gradual increase in the amount of fatigue produced by a given task.

A day's work on Tuesday is less exhausting than on Thursday or Friday. Moreover, the night's rest in very many cases is not sufficient to sweep away the fatigue of the day which it follows. For this reason a small balance of fatigue is carried over from each day to the next, and often amounts to something considerable by the end of the week. In addition, there is also a gradual diminution in the power of recovery, so that the night's rest is actually less effective at the end of a week than at its beginning. These two factors, working together, are responsible for the deterioration already described.

When Saturday arrives, the worker is as a rule quite ready for a holiday, and the week-end rest sweeps away the whole of the accumulated fatigue of the week. On Monday work is commenced with a new stock of energy. But if for any reason the week-end rest is abolished, and the worker is compelled to continue his labour without intermission, a new set of conditions is introduced. The weekly accumulations no longer being got rid of at the week's end are added together. The condition of fatigue persists, and tends to become exaggerated. From week to week and from month to month this goes on, and evidently breakdown must follow unless the process be stayed. For convenience, the process which appears to limit accumulation and prevent breakdown is called balancing. It is brought about partly by the adoption by the worker of a less strenuous attitude towards his work, and partly by an actual shortening of hours by late beginnings, early endings, and short breaks and intervals throughout the day. In this way we may explain the fact generally acknowledged in America, and gradually winning a way to acceptance here, that a too long day is extravagant, and that "shortening the hours increases the profits." This is probably true in many industries, since a long day means a slack day, with unsatisfactory output, whilst running expenses continue for a greater number of hours than when the day is short.

It has been said by some that the estimation of fatigue by physiological methods is unnecessary, since an excellent index is afforded by the output. Up to a point this is true, but it must be recognised that output is governed by other conditions besides fatigue, and it is evidently impossible to gain any knowledge of the state of the worker during rest periods and holidays through an examination of output, since at these times it does not exist. Nevertheless, the estimation of output is of value in a study of industrial conditions, and cannot profitably be neglected. Sometimes it involves only the taking of periodic readings from dials attached to machines, and under such circumstances it is easy. But it may necessitate wearisome countings of units, when it is difficult, and it becomes impossible and the method fails when several individuals work in a group, and the constitution of the group is changed at irregular intervals.

These are some of the results of our inquiry. What of their value ? Their immediate value lies in the light they shed on conditions of maximum output. Maximum output of certain kinds of military supplies has been, and now is, of vital importance to this country, and contributions to that end are of value. But when the war is over, we shall be faced by a problem of equal gravity. We shall have to settle the relations that are to exist in future between capital and labour.

Our endeavour to establish a proper understanding between master and man will be easier if it can be shown that by a shortening of hours wages may be increased, output may be augmented, and vast numbers of workers may be given time and opportunity for leisure and relaxation, and the living of their lives as reasonable beings instead of as mere machines. The old order has changed. Wages have altered. Cherished ideals of the Trade Unions have been put on one side for the sake of the country. When peace comes any attempt to go back to the old regime must lead to industrial strife. And yet industrial peace is essential in order that we may be able to deal with attempts—organised on perfect lines and lavishly financed—that will be made to steal our trade. Help given at such a time will be worth much. And it is not impossible that help may be derived from the information which has been gained. It has been shown that in certain industries maximum output—sought by capital but regarded by labour with indifference—is not necessarily bound up with long hours. It has been shown that high wages—sought by labour but not always welcomed by capital—go naturally with short hours and augmented output. It has been shown that ideal conditions for labour—short hours and high wages—go with conditions most favourable for capital—high output and low establishment charges. It has been shown that the true interests of capital and labour may be identical, and that they should work in harmony to the confusion of their foreign competitors.

The war after the war will be at least as fierce as the present struggle; it is likely to be carried on with similar unscrupulousness. Upon its results will depend the position of this country amongst the nations of the world.

At present the inquiry has been applied to comparatively few industries. Conditions differ, and what is true of one may not hold for another. We should be in a stronger position if we had accurate information with regard to the relations of fatigue, output, and wages in representative industries throughout the Empire.

